# Power Quality Control and Design of Power Converter for Variable-Speed Wind Energy Conversion System with Permanent-Magnet Synchronous Generator

**DOI:** 10.1155/2013/783010

**Published:** 2013-12-22

**Authors:** Yüksel Oğuz, İrfan Güney, Hüseyin Çalık

**Affiliations:** ^1^Department of Electrical & Electronics Engineering, Faculty of Technology, AfyonKocatepe University, Afyonkarahisar, Turkey; ^2^Department of Industrial Engineering, Faculty of Engineering, Acıbadem University Istanbul, Turkey; ^3^Electrical Department of Technical Sciences Vocational College, Istanbul University, Istanbul, Turkey

## Abstract

The control strategy and design of an AC/DC/AC IGBT-PMW power converter for PMSG-based variable-speed wind energy conversion systems (VSWECS) operation in grid/load-connected mode are presented. VSWECS consists of a PMSG connected to a AC-DC IGBT-based PWM rectifier and a DC/AC IGBT-based PWM inverter with LCL filter. In VSWECS, AC/DC/AC power converter is employed to convert the variable frequency variable speed generator output to the fixed frequency fixed voltage grid. The DC/AC power conversion has been managed out using adaptive neurofuzzy controlled inverter located at the output of controlled AC/DC IGBT-based PWM rectifier. In this study, the dynamic performance and power quality of the proposed power converter connected to the grid/load by output LCL filter is focused on. Dynamic modeling and control of the VSWECS with the proposed power converter is performed by using MATLAB/Simulink. Simulation results show that the output voltage, power, and frequency of VSWECS reach to desirable operation values in a very short time. In addition, when PMSG based VSWECS works continuously with the 4.5 kHz switching frequency, the THD rate of voltage in the load terminal is 0.00672%.

## 1. Introduction

Installation of wind energy conversion systems and rate of benefiting from the wind energy has greatly increased in last two decades. Especially in recent years, due to both a significant decrease in costs of wind power production and the technological developments in wind tribune production, contribution of wind energy in the electricity power production systems has increased rapidly [1, 2].

Variable-speed wind turbines have many advantages over fixed-speed generation such as increased energy capture, operation at maximum power point, improved efficiency, and power quality. In wind energy conversion systems, there are two operating modes of wind turbine generators (WTG) system: fixed speed and variable-speed operating modes. In order to extract maximum power from the fluctuating wind, the turbine rotor speed needs to be changed proportional to wind speed. This requires variable-speed operation. Most modern WTGs are designed for variable-speed operation [3]. Although permanent-magnetic synchronous generators (PMSG) and double-fed induction generators are widely used because of their high performance and power quality features in the variable-speed wind energy conversion systems, turbine control systems are more complicated when compared to constant speed wind energy converter systems [4–7]. The fixed-speed wind tribune applications are not generally preferred because of their low performances and as their power quality is not at a desired level. The squirrel cage asynchronous generators can be easily used both in constant speed and variable-speed tribunes as their costs are low, resistant to mechanical forces, and easily controllable. At the same time, as the squirrel cage asynchronous generators are resistant to mechanical and electrical forces and their power electronic circuits are simple, by being directly connected to the grid, they can be operated confidently [8]. On the other hand, one of the frequently used generators in the wind energy transformation system is the permanent-magnet synchronous generators.

Permanent-magnet (PM) synchronous generators are one of the best solutions for small-scale wind power plants. Low-speed multipole PMSG are maintenance-free and may be used in different climate conditions. A conventional megawatt-scale wind power plant consists of a low-speed wind turbine rotor, gearbox, and high-speed electric generator. The rotor of a typical wind turbine rotates at the speed of 15–100 rpm (150–500 rpm for small-scale wind turbines) [9]. The use of a gearbox causes many technological problems in a wind power plant, as it demands regular maintenance, increases the weight of the wind plant, generates noise, and increases power losses. These problems may be avoided using an alternative, a direct-drive low-speed PMSG.

The interaction between the wind energy conversion systems (WECS) and the grid will be an important aspect in the planning of WECS. It is essential to ensure that the grid is capable of staying within the operational limits of frequency and voltage for all foreseen combination of WECS and consumer load, and to keep, at the same time the grid transient stability [10]. The biggest problem faced during integration of the wind power production systems into existing power production systems is the quality of power. Different wind tribune types have various power quality characteristics. The power quality disturbances are power variances, vibrations, and harmonics [2, 10]. Measurement of these disturbances is standardized by IEC 61000-4-30 which define the methods for power quality parameters in 50/60 Hz A.C. power supply systems. IEC 61400-21 provides a uniform methodology to ensure consistency and accuracy in testing and assessment of power quality characteristics of grid-connected wind turbines [11–15].

There are many studies made to develop and improve the quality of power produced from the wind power production systems in the literature. Analyses for purpose of improving the power quality depending on the data obtained by observing variations in the wind power production systems have been made. These analyses contain the system stability analysis, analysis of the power converters, and stability analysis of output powers of generators in various types [16–18]. In [5], an AC-DC-AC converter system has been designed in MATLAB/Simulink for a fixed-speed wind turbine. The sinusoidal pulse width modulation (SPWM) modulator has been designed to obtain unipolar switching signals due to increased harmonic elimination features. On the other hand, a control strategy for a direct-drive stand-alone variable-speed wind turbine with a PMSG has been presented in [19]. However, a Vienna rectifier was used as PMSG generator side converter to rectify AC output voltage of PMSG wind generator and to build two equal DC voltages in [20]. The controller is capable of maximizing output of the variable-speed wind turbine under fluctuating wind. A buck-boost converter is proposed for DC chopper and the output current reference of the chopper is decided for the maximum power point tracking of wind turbine [21].

In this study, design and control of AC-DC-AC PWM power converter by using adaptive neurofuzzy controller is realized for variable-speed wind energy conversion systems at the rated power 2 MW. The inverter part of the VSWECS has been designed with a full bridge inverter, while the rectifier part has been constituted with controlled AC-DC IGBT-based PWM converter. The 11.25 Hz, 690 V, 2 MW VSWECS feeds the 50 Hz, 400 V local load or grid through an AC-DC-AC PWM converter. The 690 V, 11.25 Hz voltage is first rectified by an AC-DC IGBT-based PWM rectifier. The filtered DC voltage is applied to an IGBT two-level inverter generating 50 Hz. The IGBT inverter uses pulse width modulation (PWM) at a 1–10 kHz carrier frequency. The load voltage or grid voltage is regulated at 400 V rms by a neurofuzzy voltage regulator using abc_to_dq and dq_to_abc transformations. The first output of the voltage regulator is a vector containing the three modulating signals used by the PMW generator to generate the 6 IGBT pulses. The second output returns the modulation index. The switching frequency band of the AC-DC-AC IGBT-PWM convertor modulator has been set to 1–20 kHz, maximum THD ratio has been defined between 0.00627% and 1.05% in the harmonic analyzes performed in MATLAB/Simulink.

## 2. Variable-Speed Wind Energy Conversion System Components

### 2.1. Variable-Speed Wind Turbine

The wind energy conversion system is a complex system that converts wind energy to mechanical energy and electric energy. Output power or moment of wind turbine is defined with basic factors such as wind speed, turbine shape, and dimension. The dynamic model of a wind turbine must contain parameters defining the behavior of wind turbine. With operation of so established wind turbine, it is possible to control the performance of wind turbine to obtain desired characteristics. In respect to wind power generation, turbines having different characteristics play important role in power generation [22].

The air dynamic or wind turbine model is performed depending on the air dynamic power productivity coefficient *Cp*(*θ*, *λ*) or torque coefficient *Cq*(*θ*, *λ*), where *θ* is the blade pitch angle and *λ* is the speed rate. Output moment of the wind turbine model (air dynamic model) is determined depending on wind speed. Output power of wind turbine is multiplied by a definite gain coefficient (gear number) for stabil operation of the moment system produced depending on blade pitch angle and turbine rotor speed rate and so shaft moment is kept in desirable value. As the shaft of wind turbine and shaft of asynchronous generator is coupled with each other, the generator can be operated in desired operation speed.

With determination of input and output variables of wind turbine, expressions on relations between the input and output variables can be easily obtained. The equations defining relations between the obtained power and blade speed are given below [23]. The mechanical power in moving air flow is the flow rate of kinetic energy per second and is expressed with equation below.

The mechanical power extracted by the blades is expressed as a fraction of the upstream wind power as follows:

(1) $$\begin{array}{c} P=\frac{1}{2}\rho \cdot A\cdot V^{3}\cdot C_{p}, \end{array}$$

where *P* is mechanical power in the moving air (watt), *ρ* is air density (kg/m^3^), *A* is area swept of the rotor blades (m^2^), *V* is velocity of the air (m/sc) and *C*_*p*_ is the power coefficient.

*C*_*p*_ is the fraction of upstream wind power which is captured by the rotor blades and has a theoretical maximum value of 0.59. It is also referred to as the power coefficient of the rotor or the rotor efficiency. The power coefficient of the rotor (*C*_*p*_) is between 0.4 and 0.5 for two-blade high speed turbines. In low-speed blade turbines with more than two blades, the power coefficient varies between 0.2 and 0.4 [23]. Turbine operation conditions and obtained mechanical power can be determined by means of productive area of rotor blades, wind speed, and wind flow conditions in rotor. For this reason, turbine output power can be changed by means of change in flow conditions on rotor blades and productive area. The basis of controlling of wind energy conversion systems depends on controlling of flow condition and productive area.

The tip speed ratio *λ* is the proportion of linear speed at the tip of blade to free current wind speed and is defined with equation below [24].

Consider

(2) $$\begin{array}{c} \lambda =\frac{\omega \cdot R}{V}, \end{array}$$

where **ω** is rotor angular speed (rad/sn) and *R* is rotor blade radius (m). Obtaining of maximum power is related to rotor speed rate wind turbine operation point. Maximum wind turbine output rises to the desired value in special tip speed ratio (*λ*) and blade gap angle (*β*) values. To keep the optimal level tip speed ratio (*λ*) in all times, rotor must be rotated in high speed in high wind speeds and in low speed in low wind speeds. For productive electricity generation from wind turbines, high proportional speed turbines must be preferred [25].

For modeling of dynamic behavior of wind turbines, if the power coefficient (*C*_*p*_) that changes depending on the tip speed ratio and blade gap angle is placed in (1) and rearranged, the following equation is obtained:

(3) $$\begin{array}{c} P=\frac{1}{2}\rho \cdot A\cdot V^{3}\cdot C_{p}\left(\lambda ,\beta \right)\;\;\left(\text{Watt}\right). \end{array}$$

### 2.2. PMSG Model

The PMSG has been considered as a system which makes it possible to produce electricity from the mechanical energy obtained from the wind. In a PMSG, the field winding of the rotor is replaced by a permanent magnet. The advantages are elimination of field copper loss, higher power density, lower rotor inertia, and more robustness. The demerits are loss of flexibility of field flux control and possible demagnetization effect and its cost.

The mathematical model of the PMSG is similar to the classical synchronous machine. The PMSG is modeled with an assumption of sinusoidal-distributed windings and neglecting saturation, eddy currents, and hysteresis losses [26–28]. The parameters of the PMSG are given in the Appendix (also see Table 2). The stator *d-q* equations of the PMSM in the rotor reference frame are

(4) $$\begin{array}{c} v_{d}=R_{s}i_{d}+L_{d}\frac{{di}_{d}}{dt}-\omega_{r}L_{q}i_{q},\\ v_{q}=R_{s}i_{q}+L_{q}\frac{{di}_{q}}{dt}+\omega_{r}L_{d}i_{d}+\omega_{r}\lambda_{m}, \end{array}$$

where *R*_*s*_ is the stator winding resistance, *L*_*d*_ is the *d*-axis inductance, *L*_*q*_ is the *q*-axis inductance, *λ*_*m*_ is Amplitude of the flux induced by the permanent magnets of the rotor in the stator phases, *v*_*d*_ is the *d*-axis voltage and *vq* is the *d*-axis voltage.

In the *dq*-frame, the expression for electrodynamic torque becomes

(5) $$\begin{array}{c} T_{e}=1.5p\left[\lambda_{m}i_{q}+\left(L_{q}-L_{q}\right)i_{q}i_{d}\right]. \end{array}$$

The equation for mechanical systems for PMSG can be given as

(6) $$\begin{array}{c} \frac{d}{dt}\omega_{r}=\frac{1}{J}\left(T_{e}-F\omega_{r}-T_{m}\right),\\ \frac{d}{dt}\theta_{r}=\omega_{r}, \end{array}$$

where *p* is number of pole pairs, *T*_*e*_ is electromagnetic torque, *F* is the combined viscous friction of the rotor and load, *ω*_*r*_ is the rotor speed, *J*: the moment of inertia, *θ*_*r*_ is the rotor angular position, and *T*_*m*_ is the shaft mechanical torque.

### 2.3. Design and Control of AC-DC-AC PWM Power Converter

In the electric generation system that contains variable-speed wind tribunes, there exist three components. These are wind tribune, generator, and power converter. Power converters consist of two subsections: generator-rectifier and inverter-grid or load. In AC/DC and DC/AC power converters, different power electronic elements can be used. In this study, in the PWM power converter circuit, a two-level voltage source rectifier (VSR) and in the two-level voltage source inverter (VSI) circuit, an IGBT circuit element was used.

The grid-side inverter will draw harmonic currents inside the grid or load and because of the load impedance, harmonic voltages will occur. The voltage harmonics are in the most irregular position when the converters (rectifiers and inverters) are used. Because of the current and voltage harmonics, the quality of the obtained power will be the worst value. By using various control methods and filtering circuits, reactive power can be kept under control.

It must protect or regulate the output voltage and frequency values of the wind power generation systems according to the predetermined operation values. After the output voltage and frequency of the system are regulated according to the pre-determined values, it can be connected to the grid or load. This kind of regulation to the desired voltage and frequency values is made by means of power electronic circuit elements. During the realization of the power electronic interface circuit, any output variables of the wind power generation system (voltage, frequency, active, and reactive) must be controlled according to the determined reference value.

**Figure 1:**
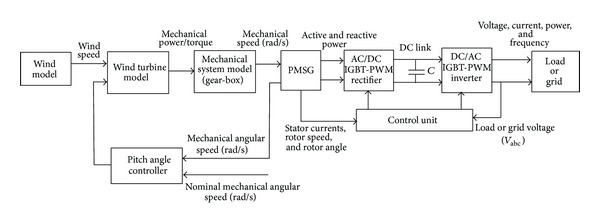
Block diagram of PMSG-based VSWECS with AC/DC/AC IGBT-PWM power converter.

In this study, the inverter of the wind power generation system is connected to the load side. DC-AC PWM inverter consists of 6 IGBT (insulated gate bipolar transistors) semiconductive elements. It can be used as a voltage source convertor. To operate the VSC, minimum DC link voltage is required. To increase the voltage phases of VSC and DC/DC converters of which voltage phases increase step by step can be defined. VSC can be operated both as an inverter and rectifier. A block diagram of wind power generation system (WPGS) with power converter is given in Figure 1.

#### 2.3.1. Control Strategy of Generator-Side AC-DC PWM Converter

In the variable-speed wind energy conversion system, there are two devices used for AC-DC conversion. One is a passive diode rectifier, which can easily convert AC to DC but cannot control its output voltage, and there may be much harmonic current in the AC source. The other conversion device is the active rectifier, which has a switching circuit topology of AC-DC power conversion. The active rectifier can convert variable frequency and variable voltage to a fixed DC voltage. In the case of PMSG variable-speed wind energy system, the power is generated at variable voltage both in frequency and amplitude. The power electronic interface is required to convert the variable voltage and frequency into a constant grid or load voltage and frequency. In this study, two back-to-back PWM power convertors are used for VSWECS. For generator-side converter, the full-bridge three-phase IGBT-PWM active rectifier is shown in Figure 1. The IGBT-PWM active rectifier is used as voltage regulator and it is controlled by using a current control algorithm [29–31]. The reference speed is calculated according to the wind tribune output power and regulated to the desired output power.

Based on the speed error, the commanded *q*-axis reference current *i*_*q*ref_ is determined through the speed controller. In this system, the following proportional-integral (PI) controller is employed as the speed controller:

(7) $$\begin{array}{c} i_{q\text{ref}}=K_{p}e_{\omega }+K_{I}\int e_{\omega }dt. \end{array}$$

**Figure 2:**
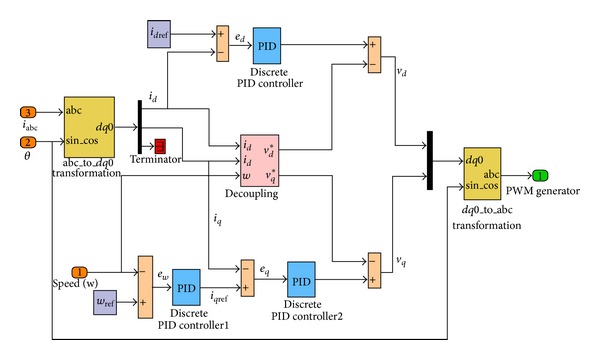
Detailed simulation block diagram of PMSG-side converter control.

While the fault between reference speed and measure speed is *e*_*ω*_, the gain parameters of the speed controller are, respectively, *K*_*P*_ and *K*_*I*_, which are proportional and integral gain parameters. *d-q*-axes voltages demanded depending on fault currents are determined by means of the current controller. In this system, the following PI controllers with decoupling terms are utilized for the current controller [31]:

(8) $$\begin{array}{c} v_{d}^{*}=K_{pi}e_{d}+K_{ii}\int e_{d}dt+\omega_{r}{Li}_{dm}, \end{array}$$

(9) $$\begin{array}{c} v_{q}^{*}=K_{pi}e_{q}+K_{ii}\int e_{q\;}dt-\omega_{r}\left({Li}_{dm}-K_{e}\right), \end{array}$$

where *K*_*Pi*_ and *K*_*Ii*_ are the proportional and integral gain coefficients of the current controller, respectively; *e*_*d*_ = *i*_*d*ref_ − *i*_*d*_ is the *d-axis* current error and *e*_*q*_ = *i*_*q*ref_ − *i*_*q*_ is the *q-axis* current error. The decoupling terms *(−*ω**_*r*_ *L*_*q*_ *i*_*q*_) and *ω*_*r*_(*L*_*d*_ *i*_*d*_ + *λ*_*m*_) are used in (8) and (9), respectively, for the independent control of the *d*- and *q*-axis currents. The commanded *dq*-axes voltages (*v*_d_*, *v*_q_*) are transformed into the physical abc quantities (*v*_a_*, *v*_b_*, *v*_c_*) and given to the PWM generator to generate the gate pulse for the PMSG-side converter. The output voltage of the PMSG is adjusted with the active rectifier [31]. In Figure 2, the detailed simulation block diagram of AC-DC, IGBT-PWM converter (active rectifier) control circuit is given.

#### 2.3.2. Control Strategy of Grid or Load-Side PWM Converter

The wind power generation systems with PMGS can generate power when either connected or not connected to the grid. In each case, the quality of power obtained from the wind generation system depends on whether the output voltage and frequency of the system is kept at desired value or not. The variable-speed power generation systems with PMSG in the literature are generally operated in a manner connected to the grid in parallel [32, 33]. The case of the variable-speed power generation system with PMSG is operated independently from the grid; its output voltage and frequency value must be kept constant within the permitted tolerance values according to the changing load situations. The basic duty of DC-AC power converter on the grid or load side is to regulate the voltage and frequency to the desired value. Voltage at load ends must be controlled in terms of both amplitude and frequency. The control structure for stand-alone control mode makes up of output-voltage controller, DC link voltage controller, dump-load resistance controller, current controller, and frequency controller in the PMSG-based VSWECS. The output-voltage controller is used to control the load (terminal) voltage during load transients or wind speed variation.

**Figure 3:**
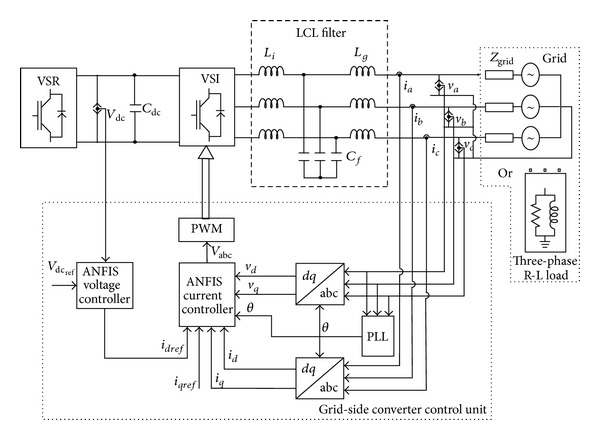
Detailed block diagram of the grid or load-side converter control.

The block diagram of the grid or load-side converter control is presented in Figure 3. The control of the grid-connected IGBT-PWM inverter is a classical one, with an inner current control loop and an output-voltage control loop, which keeps a constant value of the dc link voltage and provides the reference current for the current loop. Also, a PLL algorithm detects the phase angle of the grid, the grid frequency, and the grid voltage. The frequency and the voltage are needed for monitoring the grid conditions and for complying with the control requirements. The phase angle of the grid is required for reference frame transformations.

The voltage source converters (IGBT inverters) contain frequency harmonic components of which grid current and voltage values are high because of PWM switching. These harmonic components negatively affect the operation of sensitive or reactive loads. With the LCL filter circuit placed between the consumer or grid and voltage source converter (VSI), the effect of high frequency harmonics is drawn to a minimum. The effect of harmonics in low value switching frequencies weakens as well [34]. In Figure 3, the link of the output of the voltage source converter (IGBT-PWM inverter) to the grid by means of LCL filter is given.

*DC Link Voltage Control.* The purpose of the DC link voltage controller is to preserve the DC-link voltage at its reference value (*V*_dcref_) and to provide the reference current (*i*_*d*ref_). This controller achieved the transfer of the active power flow, drawn from the wind generator, to the utility network.

The DC-link voltage controller is designed in the continuous time domain. It consists of ANFIS (adaptive neurofuzzy inference system) controller. To achieve steady state operation the supplied DC power of the VSWECS and the ac power fed into the grid must be balanced. The dc voltage controller gives the set point of the AC power. Assuming, in this paper, that there is no power losses in the inverter and the LCL filter. The following equation is considered:

(10) $$\begin{array}{c} P_{\text{grid}}≅P_{\text{dc}}. \end{array}$$

DC side maximum link voltage is calculated with the following equation:

(11) $$\begin{array}{c} V_{\text{DC}\_\text{bağlantı}}=1,1\cdot V_{\text{faz}-\text{faz}}\cdot \sqrt{2}. \end{array}$$

To decrease the switching losses, the DC side voltage must be below the maximum voltage level. In the study, the maximum DC side link voltage is about 622 Volt. Active power difference occurred on the AC and DC side is stored in the DC-link condenser. This stored power varies depending on the DC link voltage. For this reason, size of the DC link condenser is selected according to the permitted maximum DC link voltage. Size of the DC link condenser is calculated according to the following equation [35]:

(12) $$\begin{array}{c} C_{\text{dc}\_\text{link}}=\frac{S_{N}}{{u^{*}}_{\text{dc}}\Delta u_{\text{dc}}}\cdot \frac{1}{2\omega_{n}}. \end{array}$$

In (14), *S*_*N*_ is the nominal power of voltage source converter or system, *u*_dc_* is DC side reference voltage, Δ*u*_dc_ is the difference voltage between measured DC side voltage and reference DC voltage, and *ω*_*n*_ is the angular frequency.

*Grid-Side Current Controller.* The controllers of the grid-side VSI will be obtained with respect to Figure 3. The input of the current controller is split into two terms; the reference input (*i*_*d*_*q*ref_) and the feedback input (*idq*). Output inverter current is achieved with ANFIS. The corresponding state equations are [36]:

(13) $$\begin{array}{c} {\Delta i}_{gd}=i_{d\text{ref}}-i_{d}, \end{array}$$

(14) $$\begin{array}{c} {\Delta i}_{gq}=i_{q\text{ref}}-i_{q}, \end{array}$$

(15) $$\begin{array}{c} L_{g}\frac{d}{dt}\left(i_{d\_q\text{ref}}\right)+R_{g}i_{d}-\omega L_{g}i_{q}+v_{gd}-v_{cd}=L_{g}\frac{d}{dt}\left({\Delta i}_{gd}\right), \end{array}$$

The complex valued state description in *d-q* frame indicates that the behaviour of the output LCL filter is dependent on the rotation direction of the vectors. The output LCL filter comprises two inductors (*L*_*i*_ and *L*_*g*_) and a capacitor (*C*_*f*_) connected in parallel (per phase), as shown in Figure 3. The inputs are the inverter-side voltage (*Vi*_*dq*_) and the grid-side voltage (*Vg*_*dq*_). The state and the output variables of the system are the currents through *L*_*i*_ (*i*_*idq*_) and *Lg*(*i*_*gdq*_) as well as the capacitor voltage *(Vc*_*d_q*_). The equations of voltage on inverter side in *d-q* reference axis are shown below as follows:

(16) $$\begin{array}{c} v_{d}=L_{i}\frac{d}{dt}\left(i_{d\text{ref}}\right)+R_{i}i_{d}-\omega L_{i}i_{q}+v_{cd},\\ v_{q}=L_{i}\frac{d}{dt}\left(i_{q\text{ref}}\right)+R_{i}i_{q}+\omega L_{i}i_{d}+v_{cq}. \end{array}$$

By considering Ohmic resistance values (*R*_*i*_ and *R*_*g*_) of *L*_*i*_ and *L*_*g*_ inductances of LCL filter, the above equations were obtained. Having chosen the output LCL filter values, the resonance frequency of the filter can be calculated as

(17) $$\begin{array}{c} f_{\text{res}}=\frac{1}{2\pi }\sqrt{\frac{L_{i}+L_{g}}{L_{i}L_{g}C_{f}}}. \end{array}$$

The control structure for the grid or load-connected mode of operation of the VSWECS system is implemented using (16) along with the DC voltage controller, as shown in Figure 3. The ANFIS is used to regulate the currents in the *dq* synchronous frame in the inner control loop. In this control, the IGBT-PWM converter (inverter) acts as a current-controlled voltage source. This type of control can be easily implemented in the *d-q* synchronous reference frame. This is achieved by regulating the active (*i*_*d*_) and reactive component (*i*_*q*_) of the injected current, with respect to reference values using ANFIS.

### 2.4. Frequency Control and Dump Load

Frequency of the voltage obtained in VSWECS is controlled by a separate frequency control unit. As the frequency value changes depending on the rotor speed of PMGS, when this control is not in use, no excessive fluctuation occurs in frequency. Because the wind tribune wing inclination angle is under control by the PID controller, system frequency is also kept under control. However, in this study, a frequency control unit was used as a second controller.

Input of the frequency controller is expressed with the frequency of voltage at the ends of the load. To measure the frequency of output terminal voltage at VSWESC, the three-phase-locked loop (PLL) in the SimPowerSystem Toolbox was used. The frequency value measured to determine frequency fault of the system is compared to reference frequency value and so the frequency fault is obtained. The obtained fault signal is applied to the discrete time integral element and integrated to obtain the phase. Then, the PID controller takes derivative of the fault phase.

**Figure 4:**
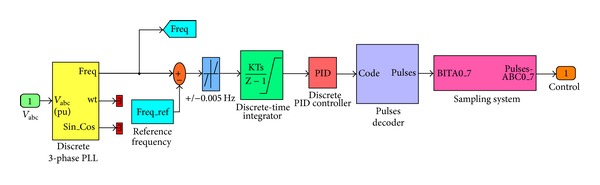
Simulation Block Diagram of Frequency Control.

As it can be seen in Figure 4, the signal obtained from the PD controller is converted to an 8-bit signal. Depending on the load situation, signals come to dump loads. Output of the frequency control unit determines the desired lump load power. The dump load consists of Gate-Turn Off (GTO) switches and the parallel connection of the 8 pieces of three-phase resistance. The dump load works according to the 8-bit binary numeric system. In consequence, there is a 28 = 256 different switching possibility. As a result of each switching possibility, load resistances have different values.

Working principle of frequency control; the generated external load control signals go to a pulse decoder. The pulse decoder has one input signal, an eight-bit output signal, and 256 different possibilities. The eight-bit signal sampling goes to the signal block and this block generates 3-phase pulse signals. External resistances are activated and inactivated by switching (GTO) according to the generated pulse control signal and so frequency of the system is kept at the desired value (50 Hz). Dump loads are widely used in voltage and the frequency control of the power generation systems are insulated from the grid and the energy distribution systems.

## 3. Simulation Results and Analysis

**Figure 5:**
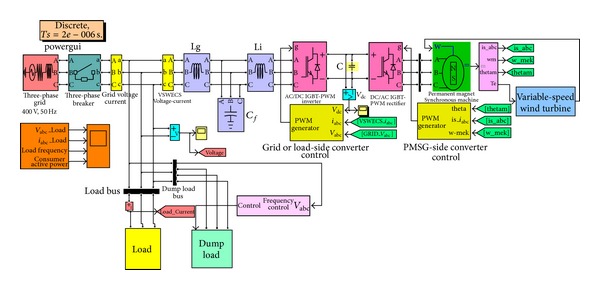
Detailed simulation block diagram of the PMSG-based VSWECS with IGBT-PWM converter connected to grid or load.

In this study, the simulations of the AC/DC IGBT-PWM active rectifier and DC/AC IGBT-PWM inverter with LCL-filter for the VSWECS are presented. Simulations have been done using MATLAB' Simulink.  Figure 5 shows the simulation model implemented in the SimPowerSystems of the MATLAB to study the performance of the PMSG-based VSWECS system operation in grid or load-connected mode. The simulation parameters of the PMSG-based VSWECS system are given in the form of tables in Appendix [37].

In the analysis of the three phase AC-DC-AC IGBT-PWM power converter designed for VSWECs with PMGS, the three-phase Ohmic-inductive load was used. The variation curves of the inverter current, inverter voltage, and terminal voltage (grid or load voltage) according to the switching frequency were examined and their total harmonic distribution rates were shown in curves. The total harmonic distribution rates were shown by using FFT solution techniques. In applications for grid/load connected IGBT-PWM converters, obtaining a high power quality and dynamic performance is important.

**Figure 6:**
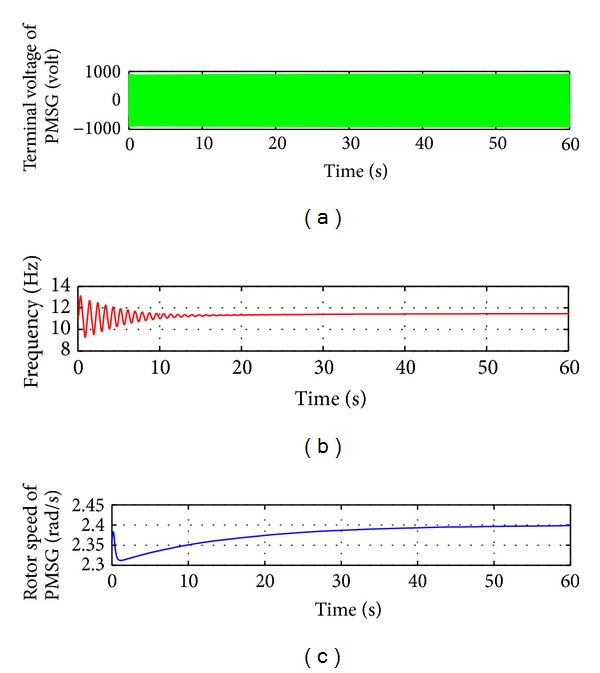
Output voltage, frequency, and rotor speed variation curves of PMSG-based VSWECS without IGBT-PWM power converter.

Before VSWECS with PMGS was connected to the three-phase AC-DC-AC IGBT-PWM power converter, it was operated with 800 kW load to determine the dynamic performance of the system. The output voltage, the frequency, and the rotor speed of PMGS were obtained (Figure 6). In Figure 6, the output voltage of PMGS is the maximum phase-phase voltage. The nominal cycle speed of PMGS is 22.5 rpm/min, frequency of output voltage is 11.25 Hz, and mechanical angular speed is 2.38 rad/sec. This is given with its variation curves in Figure 6.

The power quality of an IGBT-PWM converter depends on the switching frequency and on the line filter that is connected between the converter and the grid/load. As shown in the study, it is advantageous to use LCL-filter since sinusoidal load or line currents can be obtained even at low and moderate switching frequencies, such as 4 to 7 kHz. It is also verified that a high dynamic performance can be obtained with the LCL-filter.

Ideal harmonic phase voltage spectra of a commonly proposed PWM, 3rd harmonic injection PWM, is presented. The DC-link voltage is approximately 1.5 *U*, where *U* is the grid/line to line voltage. The modulation index (*m*) is defined by

(18) $$\begin{array}{c} m=\frac{U}{U_{\max}}, \end{array}$$

where *U*_max⁡_ is the maximum fundamental converter phase voltage. The voltage harmonics of the lowest frequency occur at frequency

(19) $$\begin{array}{c} f_{12}=\left(p\pm 2n_{1}\right). \end{array}$$

Here *p*; expresses the frequency rate defined below. *f*_mod⁡_ is the frequency modulation and *f*_1_ is the basic frequency.

Consider

(20) $$\begin{array}{c} p=\frac{f_{\mathrm{mod}}}{f_{1}},\\ m_{f}=\frac{\text{switching}\;\text{frequency}\;}{\text{fundamental}\;\text{frequency}}=\frac{f_{sw}}{f_{1}}. \end{array}$$

For high power applications (kVA) where switching losses play a major role in the overall system design, the switching frequency is usually selected between 3 kHz and 5 kHz [38].

With the simulation study, the generation power of 2 MW, 690 Volt, 11.25 Hz VSWECS was transferred to the grid or load terminals of which the working voltage was 400 volt and frequency 50 Hz via IGBT-PWM converter and LCL filter. In the study, the switching frequency range for IGBT-PWM power converter was selected between 1 kHz and 20 kHz. For various switching frequency values, THD analysis of the inverter current, the inverter current, and the grid/load voltage were made.

**Figure 7:**
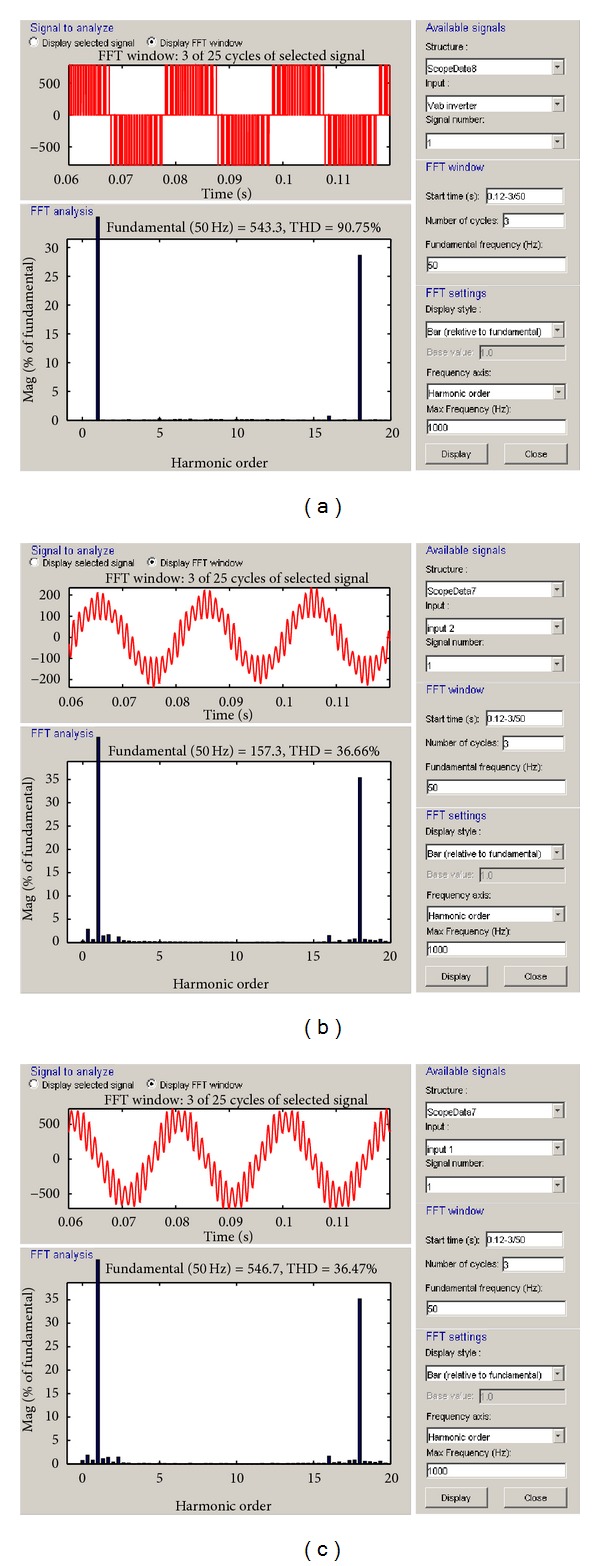
(a) The THD analyses of inverter voltage, (b) inverter current, and (c) the grid or load voltage at 1 kHz switching frequency.

In Figure 7, THD rates occurred during a 3-period process of voltage in grid/load terminals; inverter output voltage and inverter current on the inverter side of VSWECS with PMGS by using IGBT-PWM power converter in 1 kHz switching frequency are seen. In 1 kHz switching frequency, the THD rate of inverter current is 36.66%, the THD rate of inverter voltage is 90.75%, and the THD rate of terminal voltage is 36.47%. This decreases the quality and desired level of output power.

**Figure 8:**
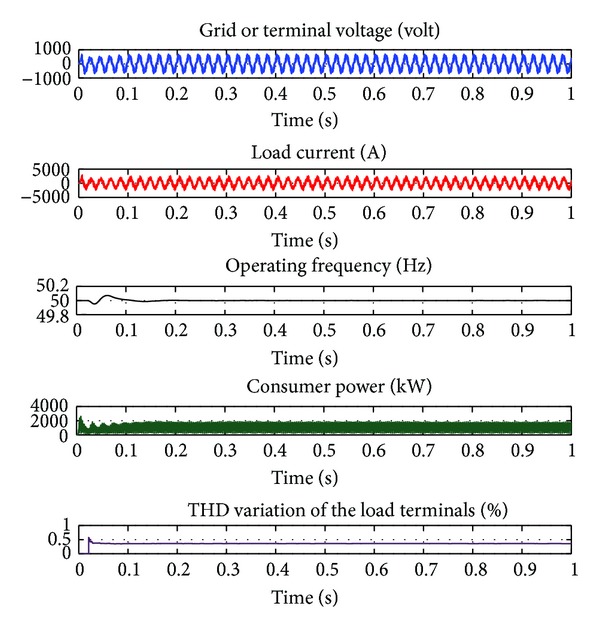
Cases of PMGS-based VSWECS containing IGBT-PWM converter feeds 800 kW consumer power in 1 KHz switching frequency, variation curves of terminal voltage, load current, operation frequency, consumer power, and THD.

Cases of PMGS-based VSWECS containing IGBT-PWM converter feeds 800 kW consumer power in 1 kHz switching frequency, variation curves of terminal voltage, load current, operation frequency, consumer power, and THD are illustrated in Figure 8. This negatively affects the harmonic distribution caused by switching frequency, the consumer, and the system itself. For this reason, in case of continuous operations of the system, the THD rate that may occur must be minimized as much as possible. In this function, determination of the switching frequency of IGBT-PWM power converter and its modulation index must be made fully.

**Figure 9:**
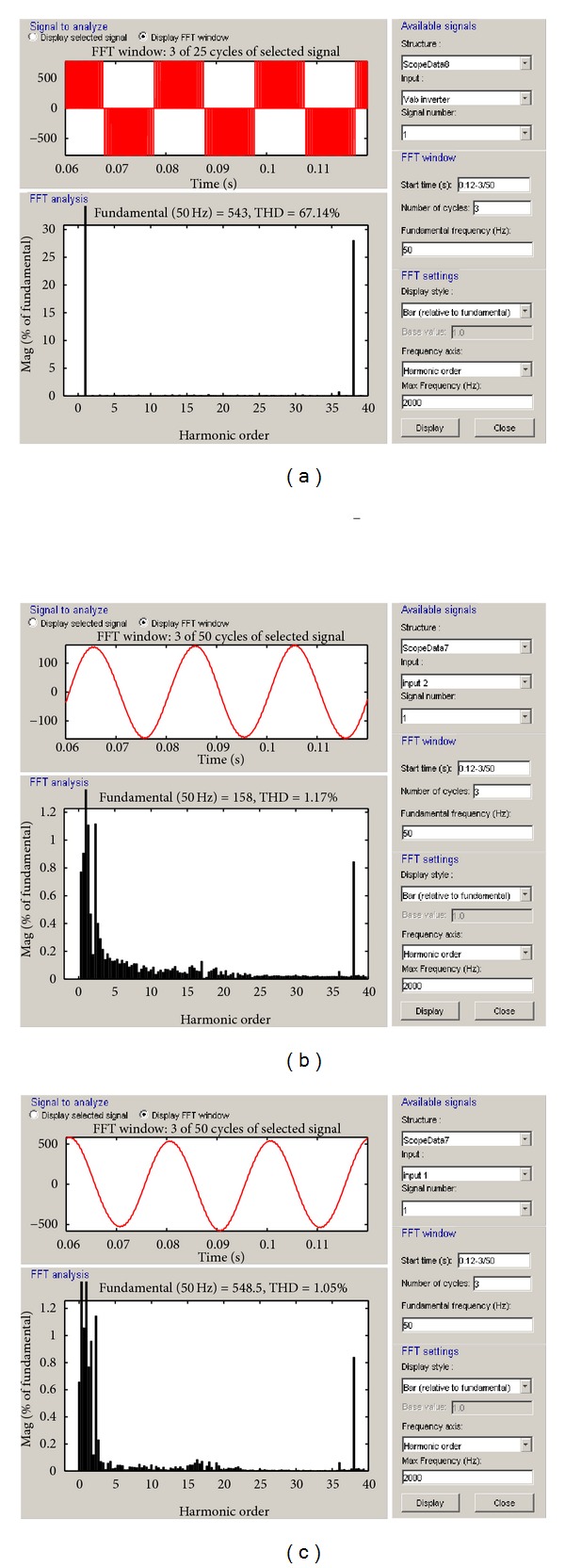
(a) The THD analyses of inverter voltage, (b) inverter current, and (c) the grid or load voltage at 2 kHz switching frequency.

**Figure 10:**
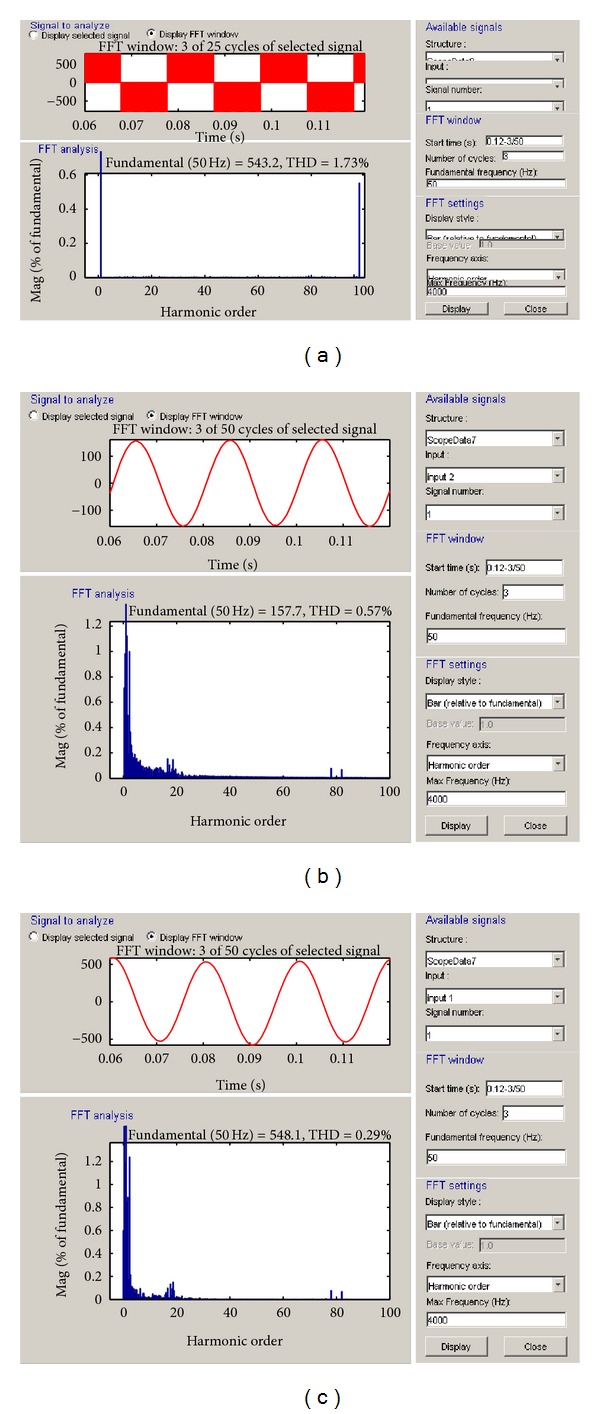
(a) The THD analyses of inverter voltage, (b) inverter current, and (c) the grid or load voltage at 4 kHz switching frequency.

**Figure 11:**
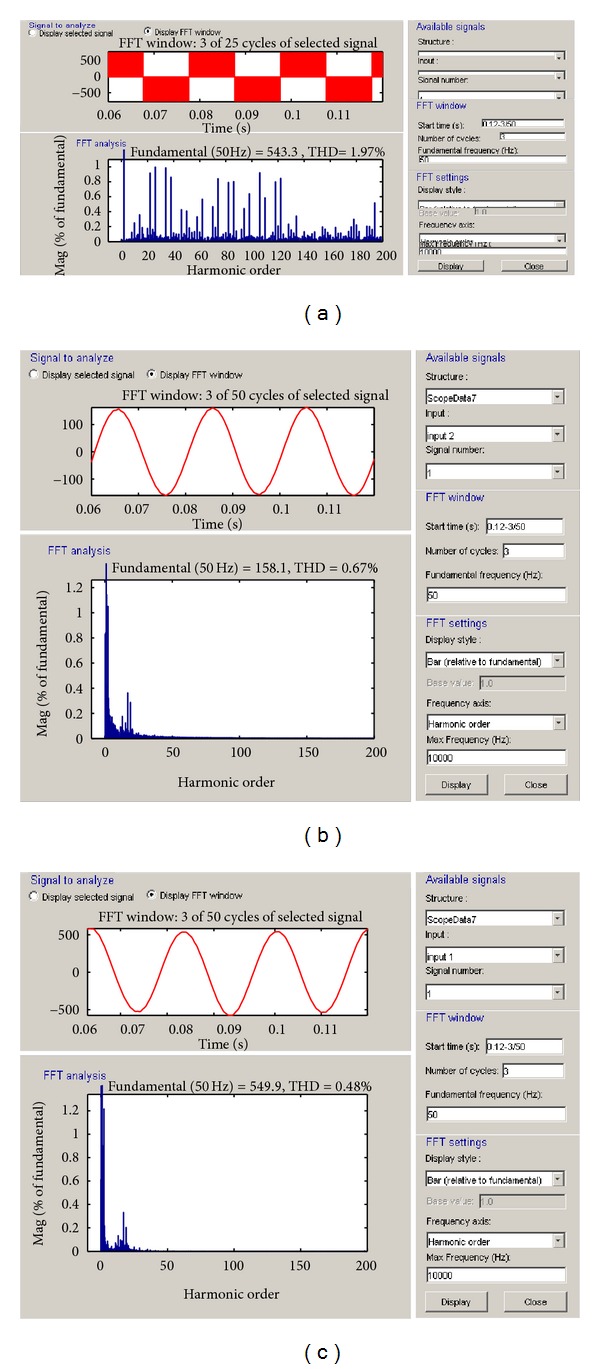
(a) The THD analyses of inverter voltage, (b) inverter current and the (c) grid or load voltage at 10 kHz switching frequency.

The THD analysis of inverter voltage, inverter current, and terminal voltage of the modelled PMSG-based VSWECS for voltage modulation index between 0.8 and 1.3 and 2, 4 and 10 kHz switching frequencies are shown, respectively, in Figures 9, 10, and 11. The variation curves in 1 kHz–20 kHz switching frequency ranges were taken but only THD rates and curves for 3 switching frequencies (2 kHz, 4 kHz, and 10 kHz) were given in this study.

**Figure 12:**
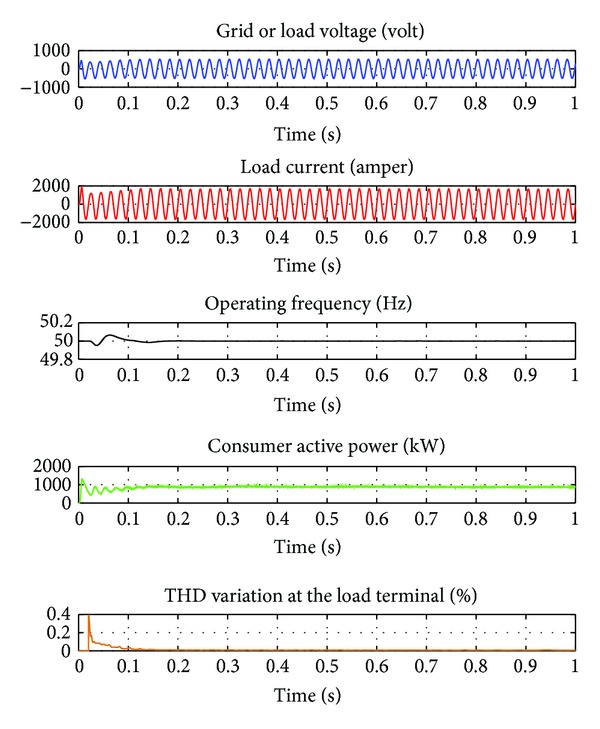
Cases of PMGS based VSWECS containing IGBT-PWM feeds 800 kW consumer power in 4 Khz switching frequency, variation curves of terminal voltage, load current, operation frequency, consumer power and THD.

In Table 1, three-periodical THD rates of inverter voltage, inverter current, and terminal voltage according to various switching frequencies are given. In Table 1, THD_*v*_ indicates the THD rate of inverter voltage, THD_*i*_ indicates the THD rate of inverter current, and THD_*g*_ indicates the THD rate of grid or terminal voltage. In the 4.5kHs switching frequency, the THD rate is 0.5%, the THD rate of inverter voltage is 1.71%, and the THD rate of terminal voltage is 0.27%. IGBT-PWM power converter with LCL output filter is designed for VSWECS with PMSG and can be used between the band width of 2 kHz–20 kHz. Because it was seen with the simulation studies that it has not affected the current and voltage values concretely or the harmonic values effectively that occurred in the switching frequency values other than 1 kHz switching values. As it can be seen in Figure 12, when PMSG-based VSWECS works permanently with the 4.5 kHz switching frequency and the THD rate of voltage in the grid or load terminals is 0.00672%. The IEEE 519 standard is a recommended practice to be used for guidance in the design of power systems with nonlinear loads and therefore should be taken into account on the design of the switching ripple filter [13]. The worst case scenario is for general distribution systems (120 V through 69000 V) with a THD < 5% for current harmonics below the 50th. The THD rates of terminal voltage and load current obtained when VSWECS works permanently are in conformity to the IEEE 519 standards.

When the load current, terminal voltage, operation frequency, and power curves drawn by a consumer in Figure 12 are examined, it is seen that no considerable deformation has occurred. No considerable deformation in voltage and frequency directly reflects to the power curve. Tolerance value range permitted for operation frequency is generally ±1%. When this tolerance value range is considered and operation frequency curve is examined in Figure 18. The obtained frequency value is between 49.5 Hz and 0.5 Hz and it is determined that at the end of about 0.15 seconds, it comes to a steady state.

## 4. Conclusions

In this study, design and control of the AC-DC-AC power converter for VSWECS with PMSG were examined. The AD-DC power converter is an active rectifier and consists of IGBT 6 pieces semiconductive circuit elements, and depending on the speed of PMSG, the rotor angle (*θ*) and stator current control of trigger circuit were made. The DC-AC power converter also consists of IGBT 6 pieces semiconductive circuit elements and depending on the grid or load side current, voltage and DC-link voltage, its control was examined. In the control of AC-DC active rectifier circuit, PID controller and in the control of DC-AC inverter circuit, ANFIS was used. The purpose in controlling the voltage source rectifier (VSR) and inverter (VSI) is to minimize the distorting effects resulting from harmonics established by switching frequencies on the current and voltage to obtain power in the desired value and quality at the output of the inverter. The grid side converter is connected to the grid/load model by means of an LCL filter of which the effect is to reduce the high frequency current ripple injected by the inverter. The grid model is represented as an ideal symmetrical three-phase voltage source. At the same time, thanks to the LCL filter connected between the AC-DC-AC power converter and the grid, an attempt was made to decrease the effect of grid voltage and load current harmonics as much as possible. From the results of the simulation study, it was determined that in case of the permanent working of VSWEC with PMSG, the THD rate of load current and terminal voltage is 0.00627%. When the studies in the literature are examined, it is seen that the obtained 0.00627% THD rate is rather at a good level. The optimal working frequency determined for VSWECS is 4.5 kHz. The switching frequency range for the desired working conditions of the AC-DC-AC IGBT-PWM power converter designed for VSWECS may be regulated between 2 kHz and 20 kHz if so is desired.

## Figures and Tables

**Table 1:** THD rates of IGBT-PWM power converter according to switching frequency values.

Switching frequency	THD_*v*_ (%)	THD_*i*_ (%)	THD_*g*_ (%)
1 kHz	90.75	36.66	36.47
2 kHz	67.14	1.17	1.05
3 kHz	67.5	0.58	0.36
4 kHz	1.63	0.57	0.29
4.5 kHz	1.71	0.5	0.27
4.6 kHz	1.58	0.58	0.28
5 kHz	1.62	0.59	0.29
6 kHz	1.65	0.61	0.31
7 kHz	1.69	0.57	0.33
8 kHz	1.68	0.59	0.32
10 kHz	1.97	0.67	0.48
15 kHz	1.741	0.55	0.50
20 kHz	2.03	0.69	0.52

**Table 2:** Model parameters of PMSG based VSWECS.

Paramaters	Values
Generator Type	PMSG, salient pole
Rated Mechanical Power	2.0093 MW
Rated Apparent Power	2.2408 MVA
Rated Line-to-line Voltage	690 V (rms)
Rated Phase Voltage	398.4 V (rms)
Rated Stator Current	1867.76 A (rms)
Rated Stator Frequency	11.25 Hz
Rated Power Factor	0.8967
Rated Rotor Speed	22.5 rpm
Number of Pole Pairs	30
Rated Mechanical Torque	852.77 kNm
Rated Rotor Flux Linkage	4.696 Wb (rms)
Stator Winding Resistance, Rs	0.73051 mΩ
*d*-axis Synchronous Inductance, Ld	1.21 mH
*q*-axis Synchronous Inductance, Lq	2.31 mH
Optimal Angle of Stator Current	19.738°

Wind turbine	Values

Type, model	W2000
Rated power	2 MW
Cut-in wind speed	4 m/s
Rated wind speed	13 m/s
Cut-out wind speed	25 m/s
Number of rotor blades	3
Rotor axis	Horizontal
Rotor diameter	76.42 m
Rotor area	4587 m²
Speed range	13 rpm–21.9 rpm
Rated speed	19 rpm

Rectifier	Values

Converter type	Two-level VSC
Modulation scheme/switching frequency	PWM/1 kHz–20 kHz

Inverter	Values

Converter type	Two-level VSC
Modulation scheme/switching frequency	PWM/1 kHz–20 kHz

DC link filter and LCL paramaters	Values

DC link filter	53.49 mF
LCL filter	1 mH, 59 *μ*F, 1 mH
